# The Potential Role of Antioxidants in the Treatment of Peripheral Arterial Disease: A Systematic Review

**DOI:** 10.3390/antiox11112126

**Published:** 2022-10-28

**Authors:** Shayan Keramat, Hiva Sharebiani, Malay Patel, Bahare Fazeli, Agata Stanek

**Affiliations:** 1 Vascular Independent Research and Education, European Foundation, 20157 Milan, Italy; 2 Department of Research and Biobanking, Buerger’s Disease Non-Governmental Organization, Mashhad 9183785195, Iran; 3 Department of Vascular Surgery, Apollo CVHF, Heart Institute, Ahmedabad 380059, India; 4 Department of Internal Medicine, Angiology and Physical Medicine, Faculty of Medical Sciences in Zabrze, Medical University of Silesia, 41-902 Bytom, Poland

**Keywords:** peripheral arterial disease, oxidative stress, antioxidant treatment, natural antioxidants, synthetic antioxidants, pro-oxidant–antioxidant balance, intermittent claudication, walking distance

## Abstract

Peripheral arterial disease (PAD) has a worldwide prevalence and is a significant cause of cardiovascular morbidity and mortality. Due to its high prevalence and higher rates of ischemic cardiovascular and lower-extremity events, its treatment is essential. Increased levels of oxidative stress cause disease. This review aimed to evaluate different studies of antioxidant treatments for PAD patients. A systematic search for relevant studies was performed on the PubMed, SCOPUS, and ScienceDirect databases, and 18 studies fulfilled the inclusion criteria. In total, 16.6% of the studies used natural antioxidants, and 83.3% used synthetic antioxidants. The reviewed studies show that natural antioxidants were completely effective in treating PAD, and synthetic antioxidants showed effective results in only 53% of the studies. A less-than-optimal pro-oxidant–antioxidant balance does not improve the symptoms of PAD. In conclusion, antioxidants in their natural forms are more effective for PAD patients, and ensuring the optimal pro-oxidant–antioxidant balance is an effective method for managing treatment with antioxidants.

## 1. Introduction

Peripheral arterial disease (PAD) is a significant cause of cardiovascular morbidity and mortality. An estimated 230 million people are affected worldwide [1]. Despite the high prevalence, PAD remains underrated, underrecognized, and undertreated [2]. Higher rates of ischemic cardiovascular and lower-extremity events, and increased prescription medication, combined with outpatient and inpatient care, can result in an increased burden on healthcare services and add to health expenses [3]. Furthermore, significant amputations can have socio-economic and psychological consequences.

PAD prevalence increases with age and affects 12–14% of the general population. Two main complications in elderly patients with PAD, and also considered to be among the leading causes of morbidity and mortality, are coronary artery disease (CAD) and cerebrovascular disease (CVD) [4]. PAD is recognized to have a greater-than-20% predictive value for coronary heart disease (CHD), mainly manifesting as a significant coronary event in the next ten years. It has been shown in patients over 50 years of age that there is a coexisting incidence of CAD in 68% of patients, while 42% have a chance of having a stroke [4,5].

Although no specific treatment has been developed for the definitive improvement of PAD, supportive therapeutic protocols are routinely used to reduce symptoms in patients with PAD. The treatment provided to a patient with PAD depends on the clinical history and the underlying disease that triggered the PAD [6]. To date, many mechanisms have been proposed for the causes of PAD. One of the most relevant causes is high levels of oxidative stress. Elevated levels of oxidants have been reported in patients with PAD, although there has been some evidence of antioxidant dysfunction in patients.

Furthermore, the balance between oxidative stress and antioxidants can be impaired in PAD [7]. High levels of oxidative stress can lead to endothelial damage, stimulate the formation of atherosclerotic plaques by LDL oxidation, and increase the levels of free radicals [8]. Therefore, some studies on PAD have considered antioxidant therapies in patients with PAD [9]. Different results have been shown for the effect of antioxidants, which can be discussed. This systematic review aimed to collect information about the impact of antioxidants in treating PAD.

## 2. Materials and Methods

### 2.1. Literature Search

In this systematic review, recommendations stated in the Preferred Reporting Items for Systematic Reviews and Meta-Analyses (PRISMA) guidelines were followed. The systematic review protocol was registered on PROSPERO (registration ID 345836). A comprehensive search of the PubMed, SCOPUS, and ScienceDirect databases was conducted from 20 September 2020 to 20 December 2021. It was found that the most important sources of antioxidants are (1) in natural and dietary forms and (2) in the form of synthetic and medicinal supplements. Diets containing vitamins E, A, and C, beta-carotene, lycopene, and selenium are the most important sources of natural antioxidants. Additionally, masoprocol, pramipexole, allopurinol, pentoxifylline, melatonin, dimethyl sulfoxide, and acetylcysteine are among the synthetic and medicinal forms. Additionally, a search was performed using relevant keywords or title headings, including ‘peripheral arterial disease’, ‘antioxidant therapy’, ‘supplementation’, ‘therapeutic effect’, and ‘complications’. Full details of the search strategy can be found in Table 1. Finally, reference lists of related review articles were manually searched.

### 2.2. Study Selection

The inclusion and exclusion criteria established for the review are indicated in Table 2. For studies to be included in the review, all the studies had to meet all the inclusion criteria. Two authors, SK and HS, separately screened the articles based on the inclusion criteria. Thus, complete reports were received for all studies that were unclear or appeared to meet the inclusion criteria. Additionally, the conflicts that occurred were resolved through analysis until an agreement was reached. When agreement could not be reached, a third reviewer (BF) participated.

## 3. Data Extraction and Quality Assessment

The reviewers independently extracted and recorded data, including the author and year of publication, population characteristics such as the sample size, antioxidant intervention (supplementation/dietary and medicinal forms), duration of follow-up, status after intervention, and quality control score as determined by the two independent reviewers.

The studies were assessed using a study design and sampling method appropriate for the research, an adequate sample size considering the prevalence of PAD, approved criteria used for the evaluation of the results, unbiased analysis of the outcomes, an adequate response rate, reporting of the statistics with confidence intervals, and detailed descriptions of the study subjects. Finally, the quality assessment showed that the selected studies could be approved in terms of all the mentioned criteria.

## 4. Results

### 4.1. Search Results

**Figure 1:**
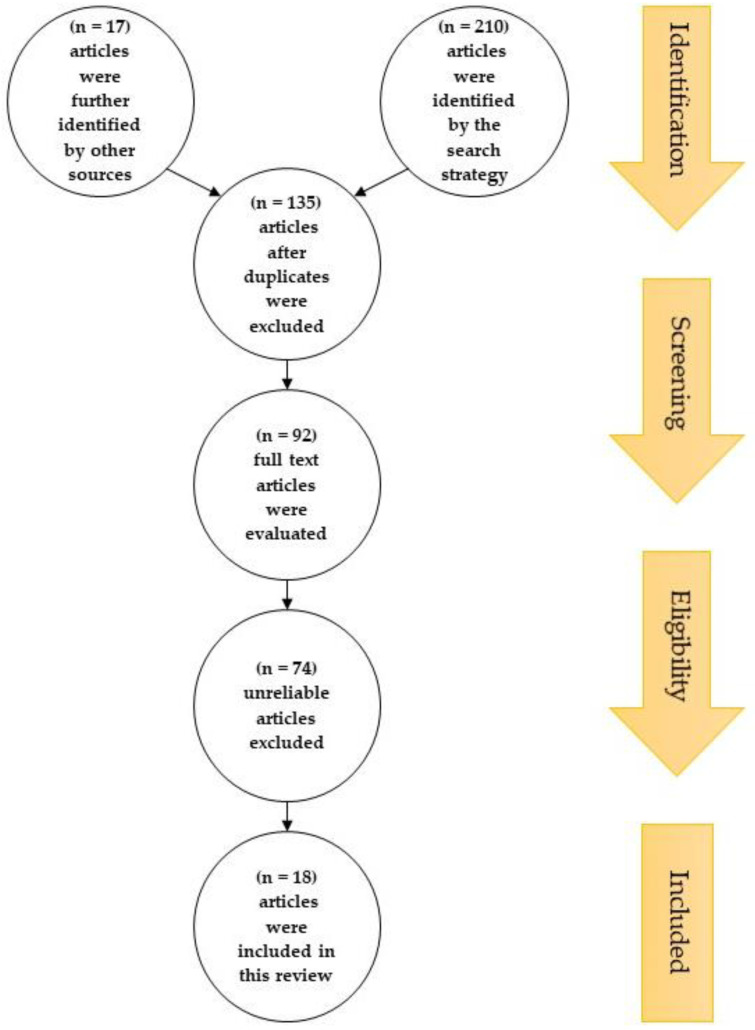
PRISMA flow diagram showing the study selection and identification. There were 210 articles; additionally, 17 articles were further identified by searching through the reference lists of relevant review. After duplicates were excluded, 135 articles were selected by title and abstract for their eligibility. From these, 92 articles were evaluated by their full texts, with 74 articles excluded due to unreliable data. Consequently, 18 articles were included in this review.

The process of choosing relevant studies is summarized in Figure 1. The search strategy identified 210 articles; additionally, 17 articles were further identified by searching through the reference lists of relevant reviews. After duplicates were excluded, 135 articles were selected by title and abstract for their eligibility. From these, 92 articles were evaluated based on their full texts, with 74 articles excluded due to unreliable study designs, patient populations, interventions, outcome measures, sample sizes smaller than 10, or no full text being available. Consequently, 18 articles were included in this review.

### 4.2. Study Characteristics

Of these 18 studies, 3 (16.6%) used natural antioxidants in the form of fruits and vegetables [10,11,12]. Fifteen (83.3%) studies used synthetic forms of antioxidants in dietary supplements and medications [13,14,15,16,17,18,19,20,21,22,23,24,25,26,27]. Of the 15 studies with the synthetic forms of antioxidants, 8 (53.3%) studies used dietary supplements [13,14,16,17,18,20,21,22] and 7 (46.6%) studies used medications [15,19,23,24,25,26,27].

Vitamins were the commonest antioxidants used, and six (33.3%) studies used vitamins as antioxidant therapy. Four (22.2%) studies used cocoa and flavonoid compounds [10,11,12,13,14,19,20,21,22,23].

Overall, no relationship between the dose and duration of antioxidant treatment was reported [10,23]. In 11 (61.1%) studies, the results for antioxidant therapy were reported to show it to be significantly effective [10,11,12,17,20,22,23,24,25,26,27], and in 7 (38.8%) studies, no significant differences from the placebo group were observed [13,14,15,16,18,19,21]. The results are summarized in Table 3.

## 5. Discussion

Oxidative stress plays a vital role in the development of PAD, but not in isolation, as other risk factors including hypertension, diabetes, smoking, hypercholesterolemia, obesity, and physical inactivity also contribute to the development of symptoms of PAD [7,28]. However, it has been shown that increased levels of oxidative stress can be an important mechanism in the pathophysiology of these risk factors, leading to increased levels of oxidized LDL (ox-LDL), increased thrombus formation, endothelial dysfunction, and the development of atherosclerotic plaques [7,8]. Therefore, it can be deduced that antioxidants may be effective in PAD treatment [29,30].

The current study was designed as a systematic review investigating the effect of antioxidant therapy in PAD patients. Two forms of antioxidants were studied—natural antioxidants (in the form of fruits and vegetables) and synthetic antioxidants (such as dietary supplements or medications).

The reviewed studies show that natural antioxidants have been thoroughly effective in treating PAD (100%), but fewer studies have used this method [10,11,12]. This beneficial effect is not consistently seen with synthetic antioxidants, and only half (53%) of the studies have shown effective results [17,20,22,23,24,25,26,27]. Based on these data, it could be assumed that antioxidants in their natural form may be more effective than synthetic forms in treating PAD. In addition, fruits and vegetables are rich sources of minerals along with antioxidants. On the other hand, the presence of minerals such as sodium, potassium, selenium, magnesium, zinc, copper, and calcium together with antioxidants and an optimal balance with them has been shown to increase the effect of antioxidants [31].

Furthermore, in 38.8% of the reviewed studies, there was no significant effect on the improvement in patients using antioxidants [13,14,15,16,18,19,21]. Furthermore, based on the reviewed studies, these also showed that the dose and duration of antioxidant therapy did not significantly affect its effectiveness.

Overall, studies on the effect of antioxidants causing improvements in PAD patients show some contradictory outcomes. On the contrary, this contradiction regarding the antioxidant effects for PAD treatment may be due to the oxidative effect of antioxidants. It has been shown that, if the level of antioxidants in the body exceeds the required level, they produce oxidative effects, and excess levels of antioxidants can lead to increased levels of oxidative stress [32]. Therefore, although the therapeutic perspective on antioxidant supplements has been optimistic, they should not be administered indiscriminately and/or excessively. The intrinsic antioxidant system in cells can prevent damage from the effects of oxidants. Therefore, checking the pro-oxidant–antioxidant balance can be an effective method for managing antioxidant treatment. The measurement of oxidant and antioxidant capacity has been shown to help in understanding the balance between them. Therefore, prescribing antioxidants may be more effective in patients after considering the pro-oxidant–antioxidant balance/ratio [32,33].

Finally, due to the multifactorial nature of PAD, using antioxidants as a definitive treatment alone may not be effective [6]. Therefore, using additional therapies according to the patient’s underlying disease (anticoagulants, antiplatelet agents, antihypertensives, antidiabetic agents, and statins) along with the appropriate amount of antioxidants seems to be more effective [6,34]. Due to the positive feedback between the initiator (cause) of pathogenesis and its consequences, there is an interaction between the oxidative stress level and thrombosis, leading to the elevation of both [35]. This is also true about the interaction between inflammation and thrombosis [36]. Therefore, interactions at multiple critical points need to be identified and addressed. Although oxidative stress is the hub of this network, we need to consider other strategic issues that can trigger a feedback loop and break the cycle started by the initiator compound. As a result, combination therapy can be more comprehensive and effective [6,35,36]. Additionally, potassium and calcium in some fruits and vegetables, such as oranges, can regulate blood pressure. Based on this, it can be assumed that natural antioxidants in fruits and vegetables can be more efficacious [31]. The review also shows a lack of further similar studies on the effectiveness of antioxidants in populations at risk of developing PAD (such as older adults and people with unhealthy lifestyles) in preventing PAD.

## 6. Conclusions

In conclusion, though there is optimism regarding using antioxidants in treating PAD, the outcomes do not support the optimism. Using antioxidants in the natural forms and vitamins in the form of fruits and vegetables is likely to be more effective for PAD patients.

Finally, two essential factors must be considered to be more effective in treating PAD. First, evaluating the optimal pro-oxidant–antioxidant balance can be an effective method for managing treatment with antioxidants. In other words, prescribing antioxidants at the appropriate dose makes antioxidants more effective and strongly suggests that custom therapies need to be devised. Secondly, using the optimal amount of antioxidants for treating the underlying disease in PAD patients, together with other agents such as anticoagulants, antiplatelet agents, antihypertensives, hypoglycemic agents, antidiabetic agents, statins, and other cholesterol-balancing drugs, is a strong recommendation.

## Figures and Tables

**Table 1:** Full details of the search strategy terms.

Terms ^1^	Search Strategy Terms
Term 1	“Antioxidants” OR “Antioxidant treatments” “Antioxidant therapy”
Term 2	“Antioxidants drugs” OR “vitamin c” OR “Ascorbic acid” OR “Vitamin A” OR “Vitamin E” OR “Lipoic acid” OR “Masoprocol” OR “Pramipexole” OR “Nitric Oxide” OR “Allopurinol” OR “Pentoxifylline” OR “Melatonin” OR “Dimethyl sulfoxide” OR “Probucol” OR “Resveratrol” OR “3-hydroxyanthranilic acid” OR Acetylcysteine OR Nicaraven OR Lodoxamide OR Mequinol OR “Hydroquinone” OR “Selenic acid” OR “Selenium” OR “Lycopene” OR “Tocopherol” OR “Rebamipide” OR “Allicin” OR “Anisodamine” OR “Bucillamine” OR “Carvedilol” OR “Pentoxifylline”
Term 3	Consumption OR “Dietary intake” OR Supplement OR Supplementation OR “Nutritional supplement”
Term 4	“Peripheral arterial disease” OR “Peripheral artery disease” OR “PAD” OR “Peripheral vascular disease” OR “PVD”, “Atherosclerosis” OR “Coronary artery disease” OR “CAD”

^1^ Term 1, 2, 3, and 4 were joined with ‘AND’.

**Table 2:** Inclusion and exclusion criteria.

Inclusion Criteria	Exclusion Criteria
Studies that are randomized controlled trials	Studies including participants at risk of PAD, including otherwise healthy smokers and hypertensive or diabetic patients
Studies with a sample size ≤ 10 participants	Studies that administer mixed nutrient supplementation where no group receives any specific antioxidant supplement alone
Studies including participants who either are healthy or have established PAD	Studies incorporating dual treatments such as exercise and supplementation
Studies that orally administer a single antioxidant intervention through supplementation or dietary or drug interventions	studies on animal models

**Table 3:** The summarized results of 18 articles that fulfilled the inclusion criteria.

	Study	Year	Sample Size (N)	Age (y)	Gender	Intervention	Form of Intervention (Natural or Synthetic)	Dose/Day	Duration	Control	Status after Intervention
1	Dalgad et al. [10]	2009	48	Not mentioned	Not mentioned	Orange and blackcurrant juice (vitamin C); orange and blackcurrant juice + vitamin E	Natural: orange and blackcurrant juice	210 mg	4 weeks	Sugar-containing reference beverage (0 mg vit c)	There were significant effects in orange and blackcurrant juice-treated patients but not in combination with vitamin E
2	Klipstein-Grobusch et al. [11]	2001	574	55–94	204 men and 370 women	Dietary β-carotene, vitamin C, and vitamin E	Natural: 70 food items in 13 food groups	Was not considered due to insufficient accuracy in the recording of the dose and duration	1 year	There were no controls in this study	In women, vitamin C intake was significantly inversely associated with the risk of PAD and lower ankle–arm systolic blood pressure Index (AAI); in men, inverse associations of PAD and AAI with vitamin E were observed; no association of αtocopherol and β-carotene with intermittent claudication
3	Woessner et al. [12]	2018	24	40–80	Not mentioned	Beetroot juice	Natural	70 mL	12 weeks	Placebo beverage	There was a significant improvement in walking distance
4	Kleijnen et al. [13]	1998	154	50–60	Men	Vitamin E	Synthetic: dietary supplement	300–900 mg	8–10 months	Placebo	There were no significant effects
5	Catalano et al. [14]	2007	210	60–70	Men	Oral antioxidant vitamins (vitamin E, vitamin C, and beta-carotene)	Synthetic: dietary supplement	600 mg vitamin E, 250 mg vitamin C, and 20 mg beta-carotene	>20 months	Placebo	There were no significant effects
6	McDermott [15]	2017	66	65	45 men and 65 women	Resveratrol	Synthetic medicinal supplement	Group 1: 500 mg (N = 22) Group 2: 125 mg (N = 22)	6 months	Placebo	There were no significant effects
7	Jepson et al. [16]	2013	78	40–70	Men and women	Allium sativum (garlic)	Synthetic: dietary supplement	800 mg	12 weeks	Placebo	There were no significant effects
8	Horsch et al. [17]	2004	619	Not mentioned	Not mentioned	Ginkgo biloba (ginkgo)	Synthetic: dietary supplement	120–160 mg	6–24 weeks	Placebo	There was a significant improvement in pain-free walking
9	Sommerfield et al. [18]	2007	425	Not mentioned	Not mentioned	Omega-3 fatty acids	Synthetic: dietary supplement	45 mg to 3 g	4 months to 2 years	Placebo	There were no significant effects
10	Curtis et al. [19]	2013	93	Not mentioned	Not mentioned	Combined isoflavone and flavan-3-ols	Synthetic combination of isoflavone and flavan-3-ols	100 mg isoflavone; 850 mg flavan-3-ols	1 year	Placebo	There were no significant effects
11	Loffredo et al. [20]	2014	20	60–70	14 men and 6 women	Cocoa	Synthetic dark chocolate (>85% cocoa)	40 g	2 h after chocolate ingestion	Milk chocolate (≤35% cocoa)	There was a significant improvement in maximal walking distance serum NOx and decreased serum isoprostanes
12	Hammer et al. [21]	2015	21	60–70	17 men and 4 women	Cocoa	Synthetic dark chocolate	50 g	2 h after chocolate ingestion	Placebo	There was no significant effect
13	McDermott et al. [22]	2020	44	70–80	Men	Cocoa	Synthetic cocoa beverage	15 g	6 months	Placebo beverage	There was a significant improvement in walking distance
14	Belch et al. [23]	2008	320	40 or more	Men and women	Antioxidant capsule contained α-tocopherol, ascorbic acid, pyridoxine hydrochloride, zinc sulfate, nicotinamide, lecithin, and sodium selenite	Synthetic antioxidant capsule combination	α-tocopherol 200 mg, ascorbic acid 100 mg, pyridoxine hydrochloride 25 mg, zinc sulfate 10 mg, nicotinamide 10 mg, lecithin 9.4 mg, and sodium selenite 0.8 m	1–5 years	Placebo	There was a significant decrease in deaths from coronary heart disease
15	Loffredo et al. [24]	2006	40	40–80	Not mentioned	Propionyl -L-carnitine	Synthetic intravenous propionyl-L-carnitine	6 mg	7 days	Placebo	There was a significant improvement in maximum walking distance
16	Loffredo et al. [25]	2007	25	40–80	Not mentioned	Propionyl-L-carnitine	Synthetic intravenous propionyl-L-carnitine	6 mg	7 days	Placebo	There was a significant improvement in the oxidative stress marker and flow-mediated dilation
17	Singh JA et al. [26]	2018	3167	75–85	Men and women	Allopurinol	Synthetic	Not enough evidence to determine	5 years	There were no controls in this study	There was a significantly lower risk of PAD in the longer allopurinol users.
18	Poggesi et al. [27]	1985	10	32–50	8 men and 2 women	Pentoxifylline	Synthetic pentoxifylline ampules; pentoxifylline tablets	100 mg pentoxifylline ampules; 400 mg pentoxifylline tablets	1–20 days	Placebo	There was a significant improvement in arterial blood flow and antithrombotic effect

## Data Availability

We used PubMed and Web of Science to screen articles for this narrative review. We do not report any data.
